# The clinical and pathological relevance of waxy casts in urine sediment

**DOI:** 10.1080/0886022X.2022.2088388

**Published:** 2022-06-22

**Authors:** Damin Xu, Jingzi Li, Suxia Wang, Ying Tan, Ying Liu, Minghui Zhao

**Affiliations:** a Renal Division, Peking University First Hospital, Institute of Nephrology, Peking University, Beijing, China; b Key Laboratory of Renal Disease, Ministry of Health of China, Beijing, China; c Laboratory of Electron Microscopy, Pathological Center, Peking University First Hospital, Beijing, China; d Peking-Tsinghua Center for Life Sciences, Beijing, China

**Keywords:** Waxy cast, urinary sediment, renal function, urinalysis, kidney disease

## Abstract

Although casts in urine may imply the underlying pathogenesis and the diagnosis, the waxy cast is poorly understood yet. We aim to investigate the association between waxy casts and clinicopathological indices. Patients undergone renal biopsy and urine sediment examination were enrolled. Waxy casts referred to those presented with a homogeneous melted wax appearance and pre-waxy casts referred to those in which one or more segments demonstrated a waxy-cast appearance. Multivariable logistic regression was used to assess the factors associated with waxy casts. In 1282 patients, the detection rate of waxy casts was 26.3%. If either waxy or pre-waxy cast was considered as a diagnostic marker for renal insufficiency (eGFR < 60 ml/min/1.73 m^2^), the sensitivity was 0.58 and the specificity was 0.88. If the only waxy cast was considered as the diagnostic marker, the sensitivity was 0.29 and the specificity was 0.97. The patients with waxy or pre-waxy casts had higher blood pressure, more proteinuria, and worse renal function. Waxy or pre-waxy cast was independently associated with eGFR (odds ratio: 0.73 per 10 mL/min/1.73 m^2^ increase, 95% confidence interval: 0.69–0.77, *p* < 0.001), proteinuria (odds ratio: 1.07 per 1 g/day increase, 95% confidence interval: 1.03–1.10, *p* < 0.001) and pathological lesions. Waxy or pre-waxy casts are closely related to impaired renal function. Their presence is a specific indicator of renal insufficiency but is not sensitive enough.

## Introduction

Urinalysis is an important technique for diagnosis and differential diagnosis, especially in kidney diseases. In addition to biochemical tests, urinalysis also needs microscopic examination to observe the formed elements in urine sediments, including cells, casts, crystals, etc. [1]. Casts are an important component in urine sediment and are associated with a variety of renal disorders. For instance, erythrocyte casts indicate glomerular diseases especially for those with glomerular cell proliferation, while renal tubular epithelial cell casts often indicate tubular injury [2]. However, waxy casts, a common element in urine sediment, are poorly investigated on their clinical and pathological relevance. Although waxy casts are considered to indicate renal insufficiency in atlases and textbooks, few studies provided convincible evidence [3]. To the best of our knowledge, only one study investigated the issue using univariable comparisons [4]. The dearth of convincible evidence and poor understanding of waxy casts may restrict the utility of urine sediment examination. Therefore, we aim to investigate the association between waxy casts and clinicopathological indices in a biopsy-proven series of patients with various kidney diseases.

## Materials and methods

### Patients

This study was performed using the data of patients who underwent percutaneous renal biopsy and urine sediment examination in our division from October 2013 to April 2015. Data including age, gender, blood pressure, proteinuria, serum creatinine (Scr), urine sediment examination, and pathological findings were obtained from the electronic database of PekingUniversity First Hospital. The levels of Scr and proteinuria closest to the day of kidney biopsy were collected. Scr was measured using Jaffe’s kinetic method. The Chronic Kidney Disease Epidemiology Collaboration equation was used to calculate eGFR [5]. Proteinuria was measured by 24-hour urine collection and the concentration of total protein was measured by a turbidimetric assay using benzethonium chloride. We excluded patients with urinary tract infection, menstruation, leucorrhea, or bleeding from genital organs, and those for whom the pathological diagnosis was uncertain or the number of glomeruli was fewer than 5 in kidney specimens. The study protocol abided by the Declaration of Helsinki and was approved by the Committee on Research Ethics of Peking University First Hospital (20171280).

### Urine specimen collection and preparation

A morning urine sample was collected for each patient on the day of the renal biopsy. Patients were instructed to clean the external genitalia before sample collection. After collection, 10 mL of urine sample in the test tube was centrifuged for 5 min at 450 g. 9.5 mL of the supernatant was removed by pipetting, and the remaining 0.5 mL of sediment left at the bottom was resuspended. Twenty microliters of resuspended sediment were transferred to a glass slide by pipetting, then covering the sample with an 18 × 18 mm^2^ coverslip. The samples would be examined within 3 h after collection.

### Urine sediment examination and classification

A phase contrast microscope (Nikon 80i) was used to observe cells, casts, and microorganisms. Polarized light microscopy was used to observe lipid and crystals. Cells were quantified as the number found in high power fields (HPF, 400×) and casts were in low power fields (LPF, 100×), respectively. Each sample was examined independently by two nephrologists blind and the average value was recorded.

**Figure 1.**
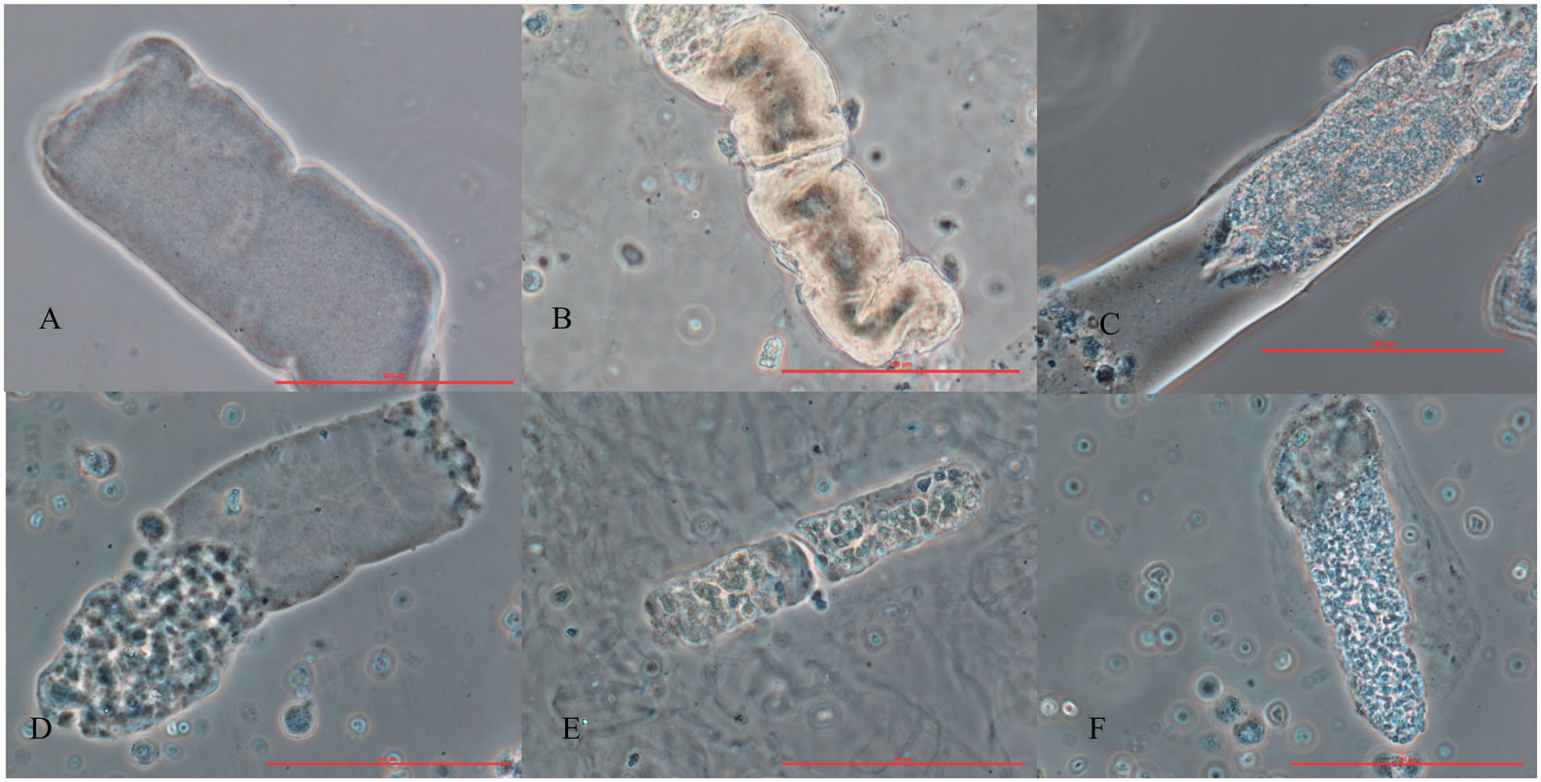
Waxy casts (A,B) and pre-waxy casts (C–F). Waxy casts scarcely contain cells or granules, characterized by high refractive index, notched and sharp margins, indented and cracked edges, and a homogeneous melted wax appearance. Pre-waxy casts contain a segment with the features of waxy casts in granular (C) or cellular casts (D–F, D: white blood cell cast; E: white blood cell and epithelial cell cast; F: red blood cell cast).

The components of urine sediment mainly included cells (RBCs, WBCs, phagocytes, tubular epithelial cells), casts (RBC casts, WBC casts, tubular epithelial cell casts, granular casts, fatty casts, waxy casts), and other formed elements. Waxy casts (Figures 1(A,B)) referred to those scarcely contain cells or granules, characterized by high refractive index, notched and sharp margins, indented and cracked edges, varied sizes, and lengths, and a homogeneous melted wax appearance [6,7]. Pre-waxy casts (Figures 1(C–F)) were defined as those cellular or granular casts in which one or more segments demonstrated the features of waxy casts.

Based on urine sediment components and urine protein level, we integrated urine sediment into four spectra as in our previous reports [8,9]: (1) dysmorphic hematuria, varying levels of proteinuria, often abundant in cells and casts, such as WBCs, phagocytes, RBC casts, WBC casts, granular casts, epithelial cell casts, which usually reflects glomerulonephritis; (2) heavy proteinuria (often nephrotic range), hyaline or fine granular casts, few cells, which indicates glomerulopathy; (3) minor proteinuria, karyocytes (mainly tubular epithelial cells, WBCs), epithelial cell casts, WBC casts, which indicate tubulointerstitial injury; (4) minor proteinuria, cells or casts occasionally, which is often observed in minor glomerular lesions, recovery stage of tubulointerstitial injury and severe glomerular sclerosis, interstitial fibrosis.

### Pathological examination and classification

Renal specimens were processed for light microscopy (LM), immunofluorescence (IF), and electron microscopy (EM) according to standard procedures. For LM examination, consecutive 3 μm paraffin-embedded sections were stained with hematoxylin-eosin, periodic acid-silver methenamine, and Masson’s trichrome. For IF examination, the 4–5 μm frozen sections were stained with fluorescein-isothiocyanate-conjugated rabbit antihuman IgG, IgA, IgM, C1q, C3, albumin, and fibrinogen antisera (Dako, USA) and examined under a fluorescence photomicroscope (Zeiss Axiophot, Oberkochen, Germany). For EM examination, renal specimens were fixed in 2.5% glutaraldehyde and post-fixed in 1% osmium tetroxide, then embedded in Epon 812 resin, ultrathin sections were mounted on metal grids and stained with uranyl acetate and lead citrate, then viewed by a transmission electron microscope (JEM-1230, Jeol, Japan). The pathological diagnoses were reviewed and confirmed by two experienced pathologists.

Pathological findings were classified into three types based on their features [10]: (1) glomerulonephritis, which is characterized by increased glomerular hypercellularity including the proliferation of indigenous cells and/or leukocyte infiltration, with or without necrosis, crescent formation; (2) glomerulopathy, such as minimal change glomerulopathy, membranous nephropathy, focal segmental glomerulosclerosis, diabetic nephropathy, and sclerotic, ischemic lesions, etc.; (3) tubulointerstitial diseases without glomerular lesion.

### Statistics

Results are expressed as mean ± standard deviation or median and interquartile range for continuous data, and as frequencies and percentages for categorical data. For continuous data, comparisons between two groups were performed using the student *t*-test or Mann–Whitney test. For categorical variables, the differences in proportions across groups were tested using Chi-squared tests or Fisher’s exact test. Receiver operating characteristic (ROC) curves, as well as the areas under the ROC curves (AUC), were used to assess the diagnostic value of waxy casts for renal insufficiency. Multivariable binary logistic regression was used to assess the factors associated with the development of waxy (or pre-waxy) casts. Because of no previous study about risk factors for waxy casts, we enrolled the candidate variables according to medical knowledge and routine practice in clinical research. Therefore, basic demographic variables (age, gender), clinical and laboratory indices related to kidney disease (blood pressure, eGFR, proteinuria), and common pathological lesions (glomerulonephritis, tubulointerstitial lesions) were enrolled in the regression model. To avoid the collinearity effect, we used mean arterial pressure instead of systolic or diastolic blood pressure as the candidate variable, we only enrolled eGFR in the regression model rather than serum creatinine. Statistical analysis was performed using the statistical software SPSS 21.0. All *p*-values were two-tailed, and the statistical significance level was defined as *p* < 0.05.

## Results

### Patient characteristics

During the study period, 1282 patients undergone renal biopsy with urine sediment examination were enrolled in the study. Of these patients, 576 were female (44.9%) and 706 were male (55.1%), with an age of 43.1 ± 16.1 (range 12–87) years. The mean blood pressure was 125.0 ± 15.3/78.3 ± 9.4 mmHg. The median serum creatinine was 83.9 (63.7–133.3) μmol/L and 321 patients (25.0%) had serum creatinine above the upper normal range (133 μmol/L). The mean eGFR was 80.1 ± 38.0 mL/min/1.73 m^2^ and 405 patients (31.6%) had eGFR lower than 60 mL/min/1.73 m^2^. The median proteinuria was 2.6 (1.0–6.0) g/24 h. According to urine sediment findings and urine protein levels, the patients were classified into four urine sediment spectra. The patients in the 1st–4th spectrum were 558 (43.5%), 599 (46.7%), 46 (3.6%), and 79 (6.2%), respectively (Table 1). According to pathological findings, the most common diagnosis was idiopathic membranous nephropathy (26.1%), followed by IgA nephropathy (24.4%) and minimal change disease (11.3%). Based on pathological features, most patients were classified into glomerulonephritis (type 1, 533 patients, 41.6%) or glomerulopathies (type 2, 716 patients, 55.8%), among whom concurrent tubulointerstitial lesions could be observed in 228 patients（112 in glomerulonephritis and 116 in glomerulopathies). Only 33 patients (2.6%) were classified as isolated tubulointerstitial lesions (Table 1).

**Table 1.** Characteristics of patients with and without waxy or pre-waxy casts.

	Total (*n* = 1282)	With waxy or pre-waxy casts (*n* = 337)	Without waxy or pre-waxy casts (*n* = 945)	*p*
Age (years)	43.1 ± 16.1	43.6 ± 17.0	42.9 ± 15.8	0.53
Gender (male)	706 (55.1%)	171 (50.7%)	535 (56.6%)	0.07
Systolic blood pressure (mmHg)	125.0 ± 15.3	129.3 ± 16.1	123.5 ± 14.7	<0.001
Diastolic blood pressure (mmHg)	78.3 ± 9.4	80.8 ± 10.3	77.4 ± 8.8	<0.001
Mean arterial pressure (mmHg)	93.9 ± 10.3	96.9 ± 11.1	92.8 ± 9.8	<0.001
Serum creatinine (μmol/L)	83.9 (63.7–133.3)	149.9 (97.3–300.6)	75.1 (59.2–99.2)	<0.001
eGFR (ml/min/1.73 m^2^)	80.1 ± 38.0	48.8 ± 35.4	91.3 ± 32.1	<0.001
Proteinuria (g/24 h)	2.6 (1.0–6.0)	3.2 (1.1–7.9)	2.4 (1.0–5.5)	0.001
Pathology				<0.001
Glomerulonephritis (*n*, %)	533 (41.6%)	208 (61.7%)	325 (34.4%)	
Glomerulopathy (*n*, %)	716 (55.8%)	107 (31.8%)	609 (64.4%)	
Isolated tubulointerstitial lesions (*n*, %)	33 (2.6%)	22 (6.5%)	11 (1.2%)	
Urine sediment				<0.001
Spectrum 1	558 (43.5%)	210 (62.3%)	348 (36.8%)	
Spectrum 2	599 (46.7%)	94 (27.9%)	505 (53.4%)	
Spectrum 3	46 (3.6%)	33 (9.8%)	13 (1.4%)	
Spectrum 4	79 (6.2%)	0	79 (8.4%)	

eGFR: estimated glomerular filtration rate. Conversion factors for units: serum creatinine in mg/dL to μmol/L, ×88.4.

### The detection rate of the waxy cast and its diagnostic value for renal insufficiency

Waxy or pre-waxy casts were found in 337 (26.3%) patients. The detection rate of waxy or pre-waxy casts in those with Scr ≤ 133 μmol/L and >133 μmol/L were 14.3% (137 patients) and 62.3% (200 patients), respectively. In urine sediment spectrum 1–4, the detection rate of waxy or pre-waxy casts were 37.6% (210), 15.7% (94), 71.7% (33), and 0, respectively. Waxy or pre-waxy casts could be seen in almost all pathological diagnoses except for amyloidosis (Supplementary Table). In pathological type 1–3, the detection rate of waxy or pre-waxy casts were 39.0% (208), 14.9% (107), and 66.7% (22), respectively.

If the presence of either waxy or pre-waxy casts was considered as a marker for renal insufficiency, the sensitivity was 0.58 and the specificity was 0.88 when renal insufficiency was defined as eGFR < 60 mL/min/1.73 m^2^, and the AUC for the ROC curve was 0.73 (95% CI: 0.70–0.76); the sensitivity was 0.62 and the specificity was 0.86 when renal insufficiency was defined as serum creatinine above the upper normal range (133 μmol/L), and the AUC for the ROC curve was 0.74 (95% CI: 0.71–0.78). If only the presence of a waxy cast was considered as a marker for renal insufficiency, the sensitivity was 0.29 and the specificity was 0.97 when renal insufficiency was defined as eGFR < 60 mL/min/1.73 m^2^, and the AUC for the ROC curve was 0.63 (95% CI: 0.59–0.66); the sensitivity was 0.34 and the specificity was 0.96 when renal insufficiency was defined as serum creatinine above the upper normal range (133 μmol/L), and the AUC for the ROC curve was 0.65 (95% CI: 0.61–0.69) (Table 2).

**Table 2.** Diagnostic value of waxy cast for renal insufficiency.

	The definition of renal insufficiency	Sensitivity	Specificity	The area under ROC curve (95% confidence interval)
Waxy or pre-waxy casts	eGFR < 60 ml/min/1.73 m^2^	0.58	0.88	0.73 (0.70–0.76)
	Serum creatinine > 133 μmol/L	0.62	0.86	0.74 (0.71–0.78)
Waxy casts	eGFR < 60 ml/min/1.73 m^2^	0.29	0.97	0.63 (0.59–0.66)
	Serum creatinine > 133 μmol/L	0.34	0.96	0.65 (0.61–0.69)

ROC curve: the area under receiver operating characteristic curve, eGFR: estimated glomerular filtration rate, conversion factors for units: serum creatinine in mg/dL to μmol/L, ×88.4.

### Comparison of clinical data and pathological features between patients with and without waxy casts

The patients with waxy or pre-waxy casts had significantly higher blood pressure (mean arterial pressure: 96.9 ± 11.1 *vs.* 92.8 ± 9.8 mmHg, *p* < 0.001), more proteinuria [3.2 (1.1–7.9) *vs.* 2.4 (1.0–5.5) g/d, *p* = 0.001] and worse renal function (eGFR: 48.8 ± 35.4 *vs.* 91.3 ± 32.1 mL/min/1.73 m^2^, *p* < 0.001) than those without. On urine sediment examination, patients with waxy or pre-waxy casts presented with more Spectrum 1 and 3, while those without waxy or pre-waxy casts presented with more Spectrum 2 and 4. According to pathological features, patients with waxy or pre-waxy casts presented with more glomerulonephritis (61.7% *vs.* 34.4%), more isolated tubulointerstitial lesions (6.5% *vs.* 1.2%) but less glomerulopathies (31.8% *vs.* 64.4%) than those without waxy or pre-waxy casts (Table 1).

We further compared the characteristics between those with waxy casts and with pre-waxy casts. Similarly, those with waxy casts had significantly higher blood pressure (mean arterial pressure: 99.3 ± 11.5 *vs.* 95.2 ± 10.5 mmHg, *p* < 0.001), higher serum creatinine [219.1 (133.2, 447.6) *vs.* 128.70 (85.0, 179.2) μmol/L, *p* < 0.001], lower eGFR (35.4 ± 30.6 *vs.* 58.7 ± 35.6 mL/min/1.73 m^2^, *p* < 0.001) and more proteinuria [3.9 (1.6–8.9) *vs.* 2.7 (1.0–6.9) g/24 h, *p* = 0.01] than those with pre-waxy casts. However, no significant difference in pathological types or urine sediment groups between the two groups.

### Assessment of factors associated with the presence of waxy casts

Multivariable logistic regression analysis revealed that the presence of waxy or pre-waxy casts was associated with eGFR, proteinuria, and pathological lesions. Each 10 mL/min/1.73 m^2^ increase of eGFR was associated with a 27% decrease in the presence of waxy or pre-waxy casts (OR: 0.73, 95% CI: 0.69–0.77, *p* < 0.001). Each 1 g increase of 24 hour-proteinuria was associated with a 7% increase in the presence of waxy or pre-waxy casts (OR: 1.07, 95% CI: 1.03–1.10, *p* < 0.001). Pathologically, glomerulonephritis (OR: 2.62, 95% CI: 1.86–3.69, *p* < 0.001) and tubulointerstitial lesions (concurrent with glomerular diseases or isolated) (OR: 1.74, 95% CI: 1.21–2.50, *p* = 0.003) were also independently associated with the presence of waxy or pre-waxy casts (Table 3).

**Table 3.** The factors associated with the development of waxy or pre-waxy casts.

	Unit of increase	Total (*n* = 1282)	
		OR (95% CI)	*p*
Age (years)	10	0.84 (0.75–0.93)	0.001
Gender (female *vs.* male)		1.67 (1.22–2.28)	0.001
Mean arterial pressure (mmHg)	10	1.04 (0.89–1.22)	0.60
eGFR (ml/min/1.73 m^2^)	10	0.73 (0.69–0.77)	<0.001
Proteinuria (g/24 h)	1	1.07 (1.03–1.10)	<0.001
Glomerulonephritis (yes *vs.* no)		2.62 (1.86–3.69)	<0.001
Tubulointerstitial lesions (yes *vs.* no)		1.74 (1.21–2.50)	0.003

eGFR: estimated glomerular filtration rate; OR: odds ratio; CI: confidence interval.

## Discussion

It is known that waxy casts in urinary sediment indicate impaired renal function, but there is no sound evidence. Only one study reported that the detection rate of the waxy cast was 13.6% in 287 patients with kidney disease [4]. In the present study involving 1282 consecutive kidney biopsy cases, the detection rate of waxy or pre-waxy casts was 26.3% in the total series and 62.3% in the renal insufficiency group (Scr > 133 μmol/L). Patients with waxy or pre-waxy casts had significantly higher Scr [149.9 (97.3–300.6) *vs.* 75.1 (59.2–99.2) μmol/L, *p* < 0.001] and lower eGFR (48.8 ± 35.4 *vs.* 91.3 ± 32.1 mL/min/1.73 m^2^, *p* < 0.001) than those without waxy or pre-waxy casts. Regression analysis showed that the risk of the presence of waxy or pre-waxy casts decreased by 27% if eGFR increased by 10 mL/min/1.73 m^2^. All these findings indicate that waxy casts are closely related to impaired renal function. Waxy or pre-waxy casts appeared in both acute and chronic renal insufficiency and in almost all pathological diagnoses, which indicates that waxy casts are not specified for certain stages or certain pathological lesions but are common in various kidney diseases with impaired renal function.

The serum creatinine test is commonly used to evaluate renal function in clinical practice [11]. The normal value of Scr is about 40–120 μmol/L, which is slightly higher in men than that in women. In addition, the normal range is slightly different due to different assay methods, which is 44–133 μmol/L in our center. In our series, 137 patients with waxy or pre-waxy casts had normal Scr (≤133 μmol/L). We screened their documents and found that 22 patients had higher Scr records (defined as Scr > 133 μmol/L or >1.5 times of the value at biopsy) before renal biopsy and eight patients had higher Scr records after renal biopsy. These findings suggest that waxy or pre-waxy casts may appear before the elevation of Scr and last for a period after renal function recovered. No elevated Scr was recorded during hospitalization in the remaining 107 patients, whose pathological diagnoses were mainly IgA nephropathy or lupus nephritis. The severity of pathological lesions may be quite heterogeneous in these diseases even in the same biopsy sample. As a result, the percentage of impaired nephrons in some cases, which contributed to waxy casts, might be not high enough to cause elevated Scr. In addition, patients with chronic kidney disease often have low Scr due to poor nutrition and low body weight. Therefore, the mild renal insufficiency might be ignored if the renal function was only evaluated by Scr, especially in those with poor nutrition. A series of more sensitive biomarkers have been proposed to detect early kidney injury, such as cystatin C, neutrophil gelatinase-associated lipocalin (NGAL), kidney injury marker-1 (KIM-1), liver-type fatty acid binding protein (L-FABP), tissue inhibitor of metalloproteinases-2 (TIMP-2) and insulin-like growth factor-binding protein 7 (IGFBP7), etc. [12,13]. In addition, an early study has shown that in patients treated with aminoglycosides, the increase of granular casts in urine was 9 days earlier than the increase of Scr [14]. In our study, waxy casts appeared even in the patients with normal Scr, who needed more close monitoring for potential renal injury. Patients with pre-waxy casts had lower Scr than those with waxy casts [128.7 (85.0,179.2) *vs.* 219.1 (133.2,447.6) μmol/L, *p* < 0.001], this finding suggests that pre-waxy casts may be a marker for mild renal insufficiency.

In our study, waxy casts rarely appeared alone but were often accompanied by other types of casts, such as RBC casts, WBC casts, granular casts, and renal tubular epithelial casts. Moreover, one or more waxy segments could present in almost all other types of casts (RBC casts, WBC casts, granular casts) (Figures 1(C–F)), which are named pre-waxy casts in our study. It is not known whether these pre-waxy casts are a simple mixture of two types of casts or derivatives from other casts. In fact, the origin and composition of waxy casts have not yet been fully understood. An early report observed that waxy casts had a surface that was slightly irregular and broken up into ‘plates’, which overlaid a fibrillar internal meshwork similar to that observed in hyaline casts. This finding supported the hypothesis that waxy casts derive from the degeneration of other types of casts [15]. On the contrary, a study about urine sediment published in abstract format [16] reported that they observed T-H glycoprotein, which is previously considered as a matrix component in various types of casts [17–19], in all hyaline, hyaline-granular, and mixed casts, but was not in waxy casts. Due to the lack of in-depth research and sufficient evidence, the composition of waxy casts is still a mystery and needs further exploration.

All the diagnoses in our series were confirmed by renal biopsy. The pathological findings almost covered various kidney diseases, as shown in the Supplementary Table. In contrast to a previous report that 4 (44.5%) of the nine renal amyloidosis cases had waxy casts [4], in our series, the waxy or pre-waxy cast was not detected in all 10 renal amyloidosis cases (three cases with Scr > 133 μmol/L at renal biopsy), more amyloidosis cases are needed to investigate the association between waxy casts and amyloidosis.

In our analysis, older age seemed negatively associated with the presence of waxy casts, which could not be explained by medical knowledge. Despite the statistical significance, the result should be confirmed by further studies. Glomerulonephritis and tubulointerstitial lesions were the risk factors for the presence of waxy casts, however, they might not be independent variates, because their severity might be parallel in some cases and the collinearity might exist in the regression model. So we performed collinearity diagnostics and the variance inflation factor was low (1.32 for glomerulonephritis and 1.36 for tubulointerstitial lesions), which might be due to the low rate (18.3%) of concurrent tubulointerstitial lesions in glomerular diseases (i.e., glomerulonephritis and glomerulopathies). In addition, two common markers for kidney injury, proteinuria and the decrease of eGFR were also the risk factors for the presence of waxy casts, which suggested that renal injury could also be reflected in urinary sediment. Therefore, urinary sediment examination should be performed routinely to detect waxy or pre-waxy casts in patients with kidney diseases.

The present study represents the largest series on waxy casts and provides sound evidence on the association between waxy casts and renal function. We are also aware of the potential limitations. Firstly, we used a single urine sample to examine waxy casts, which might underestimate the detection rate. Secondly, it still needs investigation whether different pre-waxy casts (e.g., pre-waxy casts with granular casts, pre-waxy casts with WBC casts, pre-waxy casts with epithelial cell casts, etc.) have different clinical or pathological implications. Unfortunately, the detailed features of pre-waxy casts were not recorded in the urine sediment report and they will be recorded in the future. Thirdly, as a single-center study, the characteristics of the series may be different from other centers, and the conclusion needs to be further verified in multi-center studies.

In conclusion, waxy or pre-waxy casts in urinary sediment are closely related to impaired renal function and are indicators of renal injury. Waxy or pre-waxy casts can be observed in various kidney diseases. Urine examination, as a time-honored, simple, and useful technology, should be made full use of in clinical practice.

## Supplementary Material

Supplemental Material
