# Supplementary material for: The clinical and pathological relevance of waxy casts in urine sediment
Source: Ren Fail. 2022 Jun 22;44(1):1038–44. doi: 10.1080/0886022X.2022.2088388 (PMC9246008; doi:10.1080/0886022X.2022.2088388)
Supplement: Supplemental Material [file IRNF_A_2088388_SM2837.pdf]

Supplementary table. Pathological findings and the frequencies of waxy casts

	Number (n, %[a])	Proteinuria (g/24h)	eGFR (ml/min/1.73m^2)	eGFR<60 (n, %[b])	With waxy or pre-waxy casts (n, %[b])
**Glomerulonephropathy**					
Minimal change disease	145 (11.3%)	3.4 (0.6-8.4)	97.5 ± 32.3	20 (13.8%)	19 (13.1%)
Focal segmental glomerulosclerosis	52 (4.1%)	9.7 (4.0-14.9)	72.1 ± 42.2	21 (40.4%)	20 (38.5%)
Idiopathic membranous nephropathy	335 (26.1%)	4.1 (2.0-7.2)	94.3 ± 26.7	38 (11.3%)	29 (8.7%)
Atypical membranous nephropathy	58 (4.5%)	5.1 (2.2-8.8)	104.7 ± 36.8	9 (15.5%)	8 (13.8%)
HBV/HCV associated membranous nephropathy	23 (1.8%)	3.7 (2.1-7.5)	95.1 ± 28.4	2 (8.7%)	2 (8.7%)
GVHD associated membranous nephropathy	1 (0.1%)	4.6	131.4	0	0
Diabetic nephropathy	25 (2.0%)	5.9 (3.0-10.2)	43.5 ± 28.8	17 (68.0%)	10 (40.0%)
Amyloidosis	10 (0.8%)	5.6 1.9-8.1)	75.4 ± 34.6	3 (30.0%)	0
MGRS (except amyloidosis)	15 (1.2%)	3.1 (1.4-8.6)	43.4 ± 21.3	12 (80.0%)	6 (40.0%)
Ischemic nephropathy	19 (1.5%)	0.4 (0.02-1.6)	45.3 ± 25.3	16 (84.2%)	4 (21.1%)
Obesity-associated glomerulomegaly	8 (0.6%)	2.3 (0.4-3.9)	73.6 ± 32.3	3 (37.5%)	2 (25.0%)
Alport syndrome	6 (0.5%)	0.9 (0.4-4.0)	86.7 ± 30.1	1 (16.7%)	1 (16.7%)
Collagen Ⅲ glomerulopathy	1 (0.1%)	3.8	102.1	0	0

Information Classification: General

Fabry disease	1 (0.1%)	1.1	124.8	0	0
Thin basement membrane nephropathy	9 (0.7%)	0.6 (0.1-1.1)	88.2 ± 27.9	1 (11.1%)	1 (11.1%)
Thrombotic microangiopathy	8 (0.6%)	1.4 (0.4-2.6)	45.1 ± 43.4	7 (87.5%)	6 (75.0%)
**Glomerulonephritis**					
IgA nephropathy	313 (24.4%)	1.2 (0.7-2.8)	76.9 ± 34.5	108 (34.5%)	97 (31.0%)
IgA vasculitis (HSP nephritis)	39 (3.0%)	2.2 (1.3-3.6)	90.5 ± 43.2	11 (28.2%)	19 (48.7%)
Mesangioproliferative glomerulonephritis	29 (2.3%)	0.8 (0.4-1.8)	64.9 ± 35.8	16 (55.2%)	11 (37.9%)
Crescentic glomerulonephritis	35 (2.7%)	2.7 (0.9-4.4)	17.5 ± 15.0	34 (97.1%)	28 (80.0%)
ANCA associated systemic vasculitis (non-crescentic)	14 (1.1%)	0.7 (0.5-1.3)	27.3 ± 18.4	13 (92.9%)	7 (50.0%)
Endocapillary proliferative glomerulonephritis	5 (0.4%)	3.7 (2.8-6.2)	29.3 ± 18.5	5 (100.0%)	5 (100.0%)
Lupus nephritis	72 (5.6%)	3.5 (1.8-7.4)	79.8 ± 41.1	24 (33.3%)	34 (47.2%)
C3 glomerulopathy	9 (0.7%)	2.8 (1.5-5.3)	56.5 ± 36.5	6 (66.7%)	4 (44.4%)
MPGN (except C3 nephropathy)	17 (1.3%)	6.4 (2.1-9.3)	64.7 ± 30.3	8 (47.1%)	7 (41.2%)

Information Classification: General

**Tubulointerstitial disease**	33 (2.6%)	0.8 (0.2-1.1)	31.5 ± 23.3	30 (90.9%)	22 (66.7%)
Total	1282 (100%)	2.6 (1.0-6.0)	80.1 ± 38.0	405 (31.6%)	343 (26.8%)

HBV: hepatitis B virus; HCV: hepatitis C virus; GVHD: graft-versus-host disease; MGRS: monoclonal gammopathy of renal significance; HSP: Henöch-Schonlein purpura; MPGN: membranoproliferative glomerulonephritis.

a. The percentage in all 1282 patients

b. The percentage in each pathological diagnosis.
